# Supplementary material for: Anaerobic thiosulfate oxidation by the Roseobacter group is prevalent in marine biofilms
Source: Nat Commun. 2023 Apr 11;14:2033. doi: 10.1038/s41467-023-37759-4 (PMC10090131; doi:10.1038/s41467-023-37759-4)
Supplement: Supplementary file 1 — Supplementary Information [file 41467_2023_37759_MOESM1_ESM.pdf]

Supplementary Information

Ding W et al.,

Anaerobic thiosulfate oxidation by the *Roseobacter* group is prevalent in marine biofilms

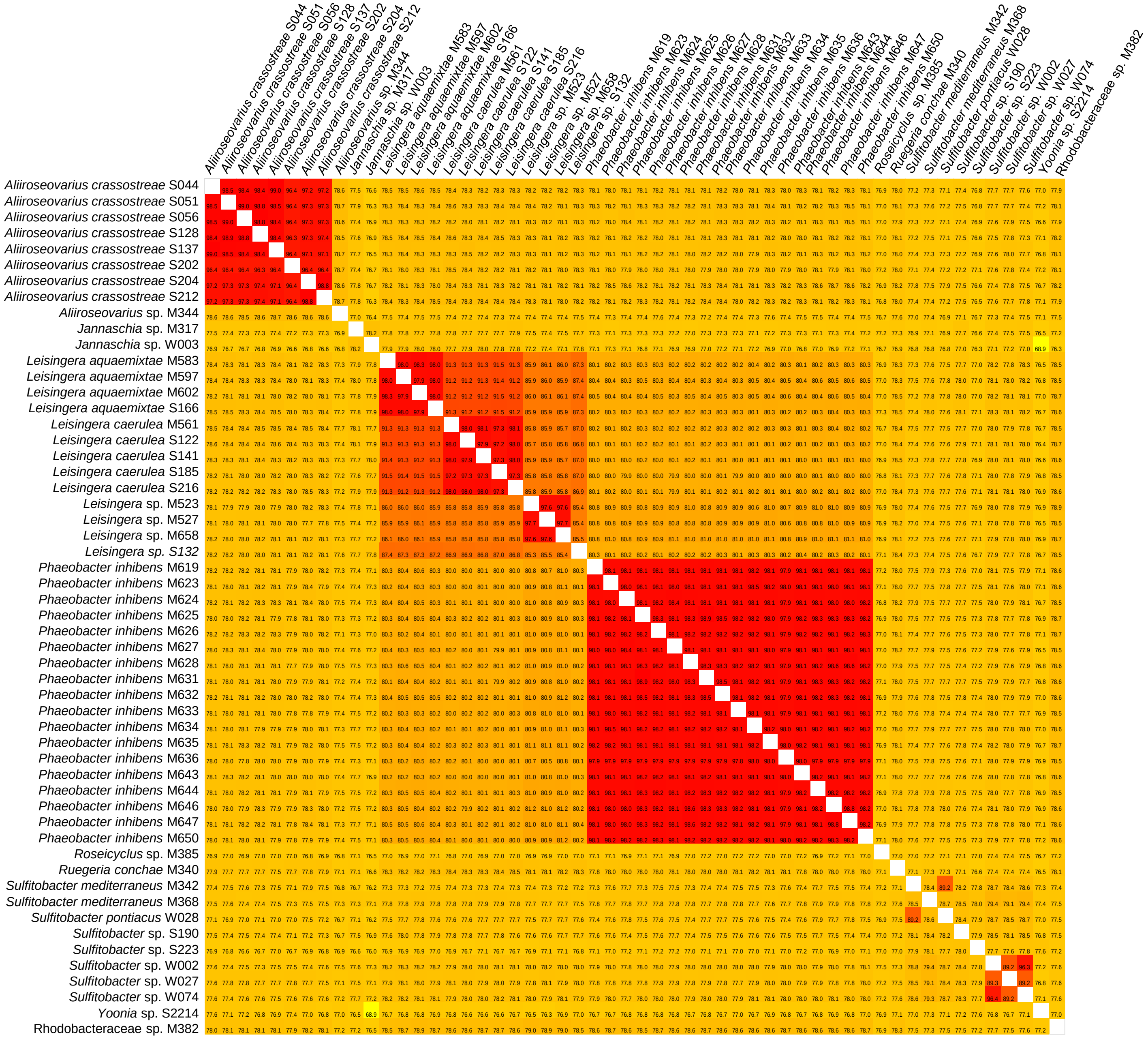

Supplementary Fig. 1 Pairwise average nucleotide identity comparison between the 54 biofilm *Roseobacter* strains. The average nucleotide identity values were calculated using chromosomes as queries, and strain-level dissimilarities (> 99%) were obtained for all the pairwise comparisons.

	**Rhodobacteraceae sp. M382**	*Ruegeria pomeroyi* DSS-3	*Ruegeria* sp. AD91A	*Ruegeria* sp. THAF33
**Rhodobacteraceae sp. M382**		78.6	78.1	78.2
Ruegeria pomeroyi DSS-3	78.6		78.9	79.4
Ruegeria sp. AD91A	78.1	78.9		91.0
Ruegeria sp. THAF33	78.2	79.4	91.0	

Supplementary Fig. 2 Average nucleotide identity comparisons of Rhodobacteraceae sp. M382 with its close relatives. The ANI values were calculated using chromosomes as queries.

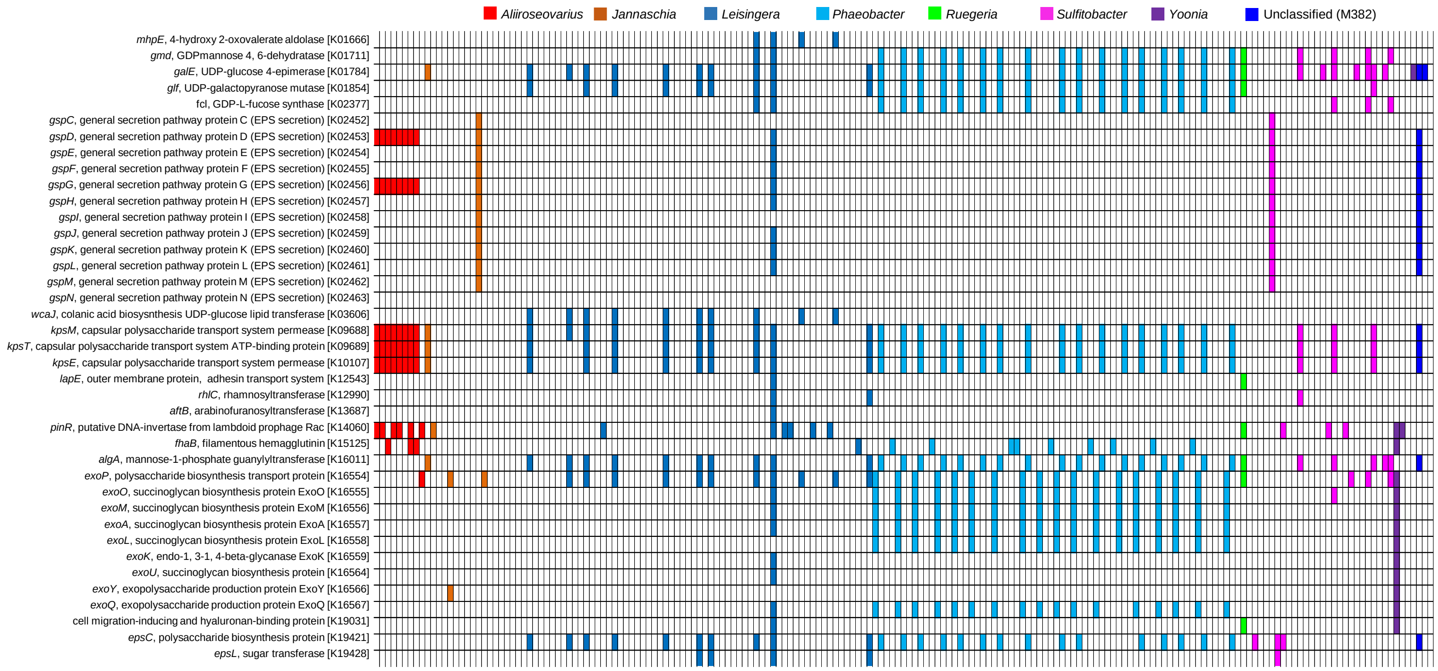

Supplementary Fig. 3 The presence of genes involved in biofilm formation in plasmids of the biofilm *Roseobacter* strains. Genes responsible for the secretion of extracellular polysaccharides, biosynthesis of mannose, rhamnose, and polysaccharides, regulation of polysaccharide biosynthesis (e.g., DNA-invertase), and surface addition (e.g., filamentous hemagglutinin) were identified. The color is coded by bacterial group and a colored box indicates the presence of the respective gene.

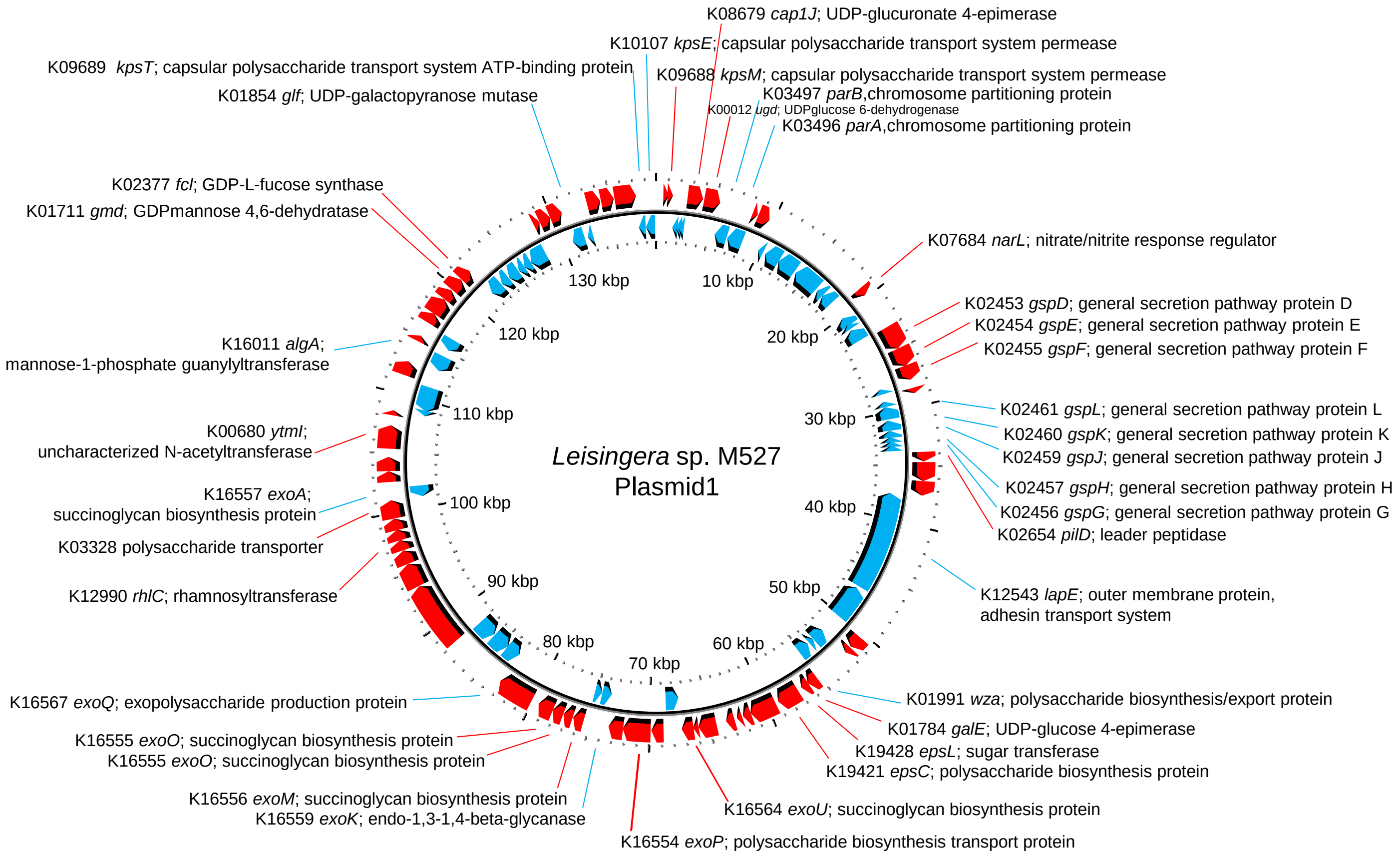

Supplementary Fig. 4 Map of a plasmid from *Leisingera* sp. M527. Gene functions were annotated by BLASTp searching against the KEGG database. Gene orientations were indicated by arrows colored by blue or red. Many of the annotated genes, such as *epsLC* (exopolysaccharide biosynthesis), *gspDEFLKJHG* (polysaccharide secretion), *pilD* (pilus assembly) are known to be involved in biofilm formation and thus this probably a typical biofilm plasmid.

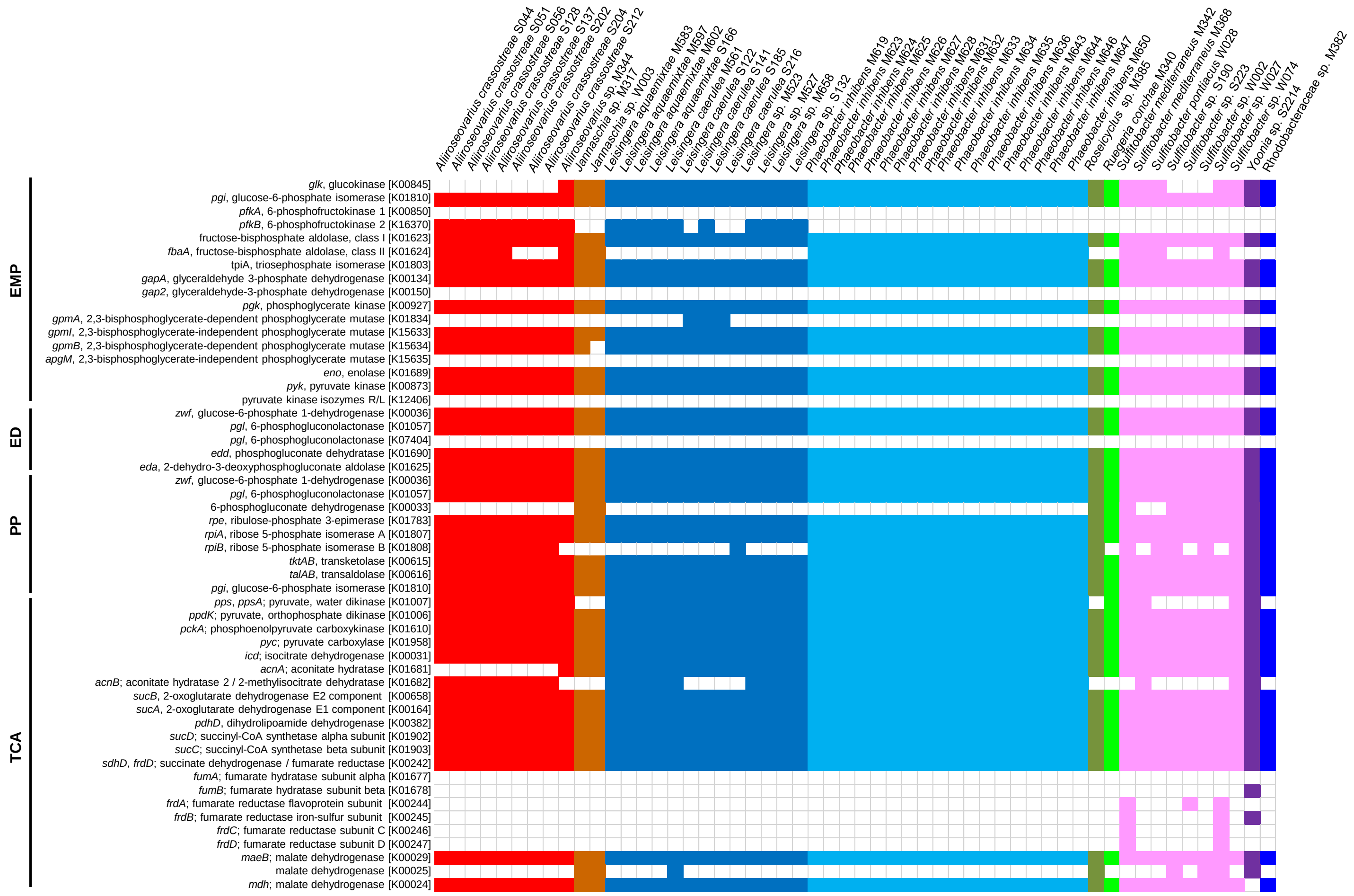

Supplementary Fig. 5 The presence of central carbon metabolism genes in the biofilm *Roseobacter* genomes. Gene functions were annotated by BLASTp searching against the KEGG database. The color is coded by bacterial group and a colored box indicates the presence of the respective gene. TCA, tricarboxylic acid; ED, the Entner-Doudoroff pathway; PP, the pentose phosphate pathway; EMP, the Embden-Meyerhof-Parnas pathway.

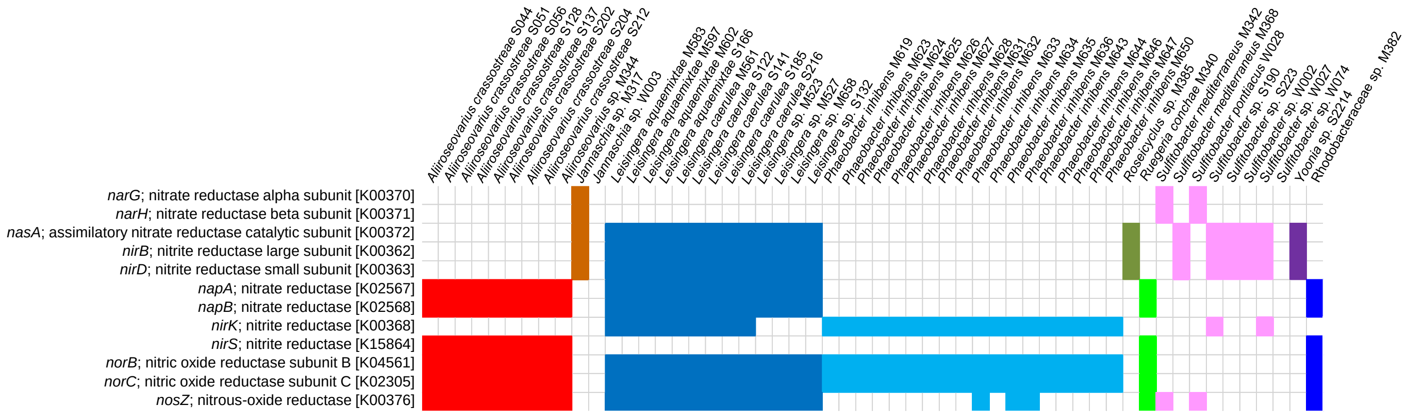

Supplementary Fig. 6 The presence of nitrogen metabolism genes in genomes of the biofilm *Roseobacter* strains. Gene functions were annotated by BLASTp searching against the KEGG database. The color is coded by bacterial group and a colored box indicates the presence of the respective gene.

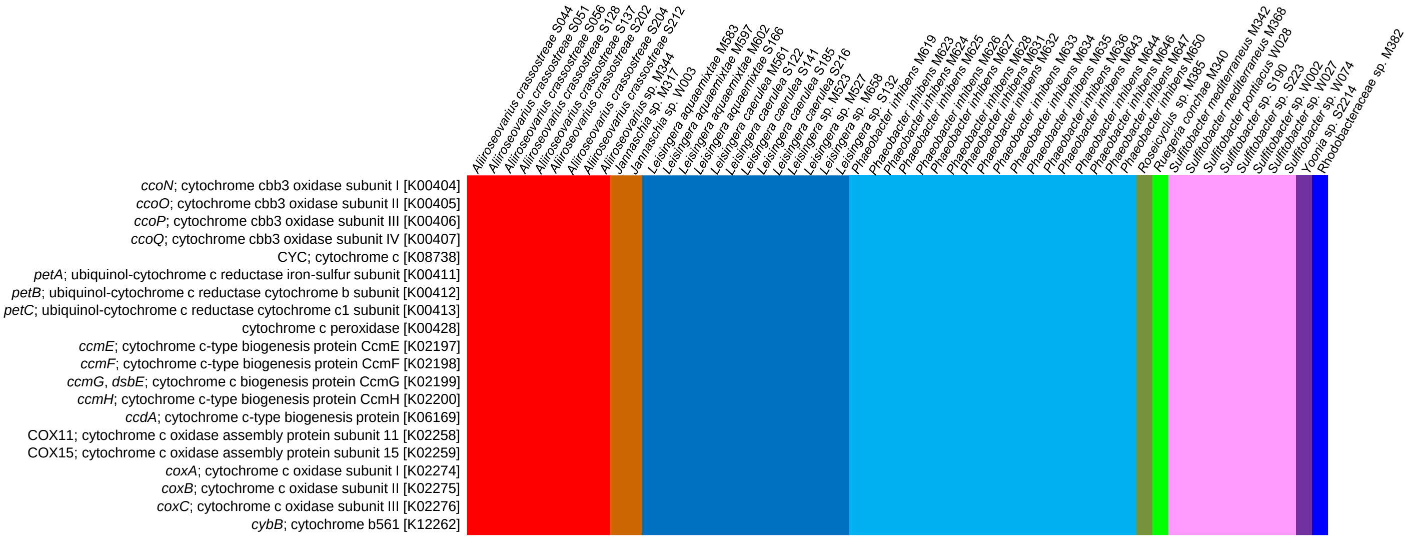

Supplementary Fig. 7 The presence of cytochrome related genes in genomes of the biofilm *Roseobacter* strains. Gene functions were annotated by BLASTp searching against the KEGG database. The color is coded by bacterial group and a colored box indicates the presence of the respective gene.

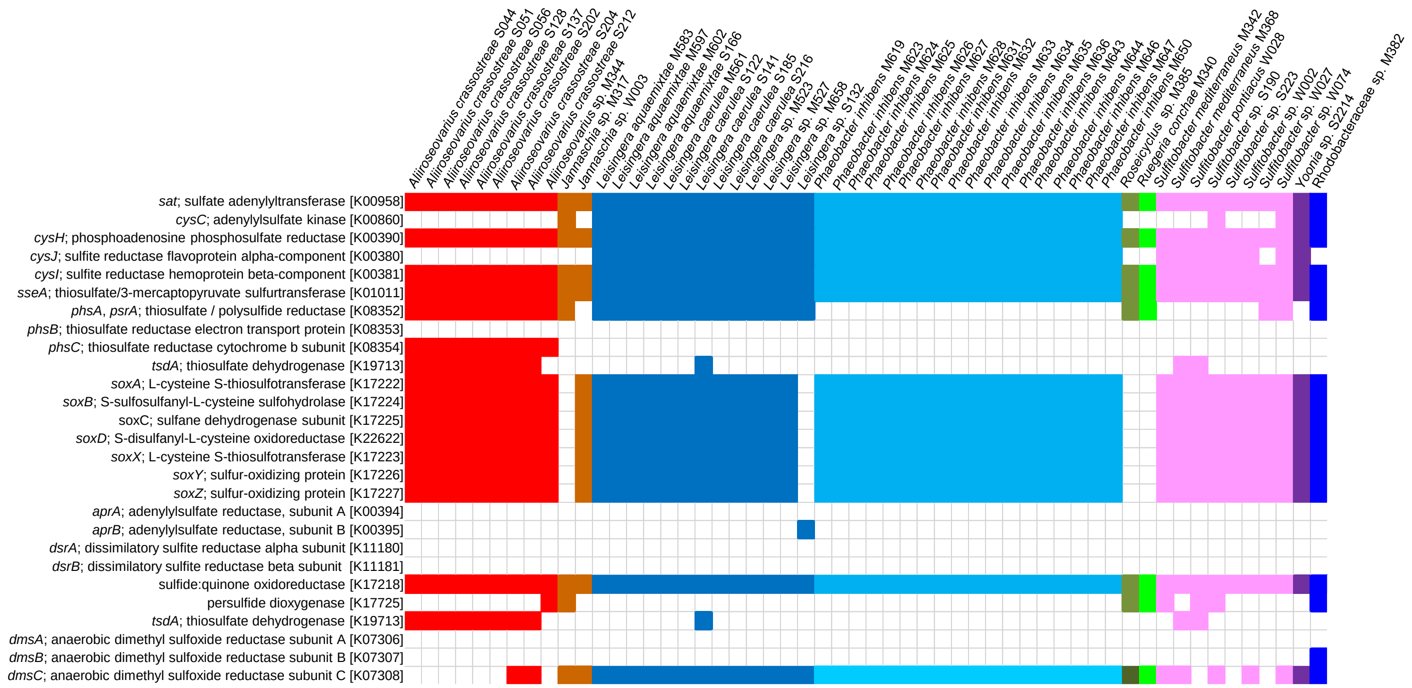

Supplementary Fig. 8 Distribution of sulfur metabolism genes in genomes of the biofilm *Roseobacter* strains. Genes were annotated by BLASTp searching against the KEGG database. The color is coded by bacterial group and a colored box indicates gene presence while an empty box indicates gene absence.

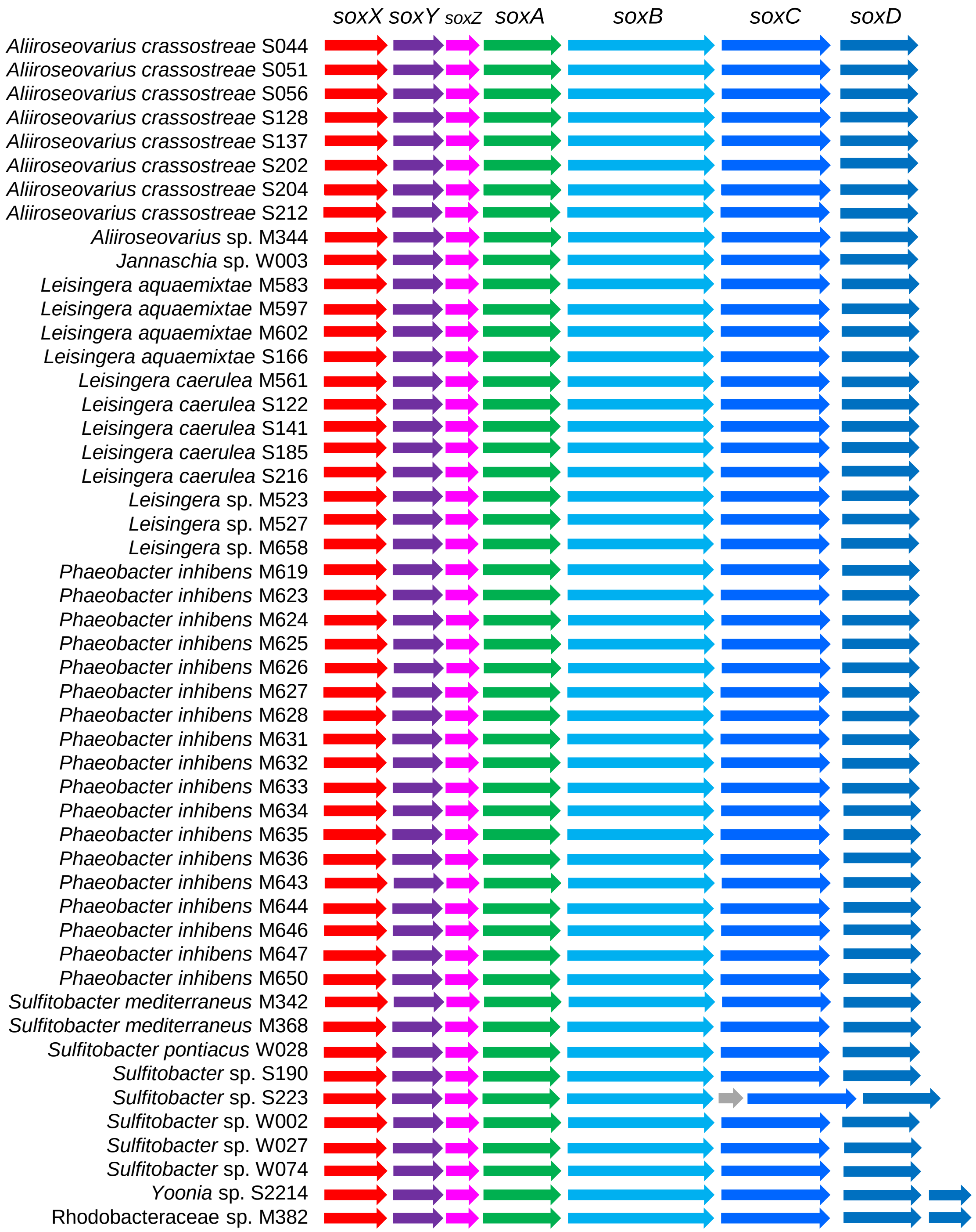

Supplementary Fig. 9 The *sox* gene operon in genomes of the biofilm *Roseobacter* strains. The seven *sox* genes were indicated by different colors.

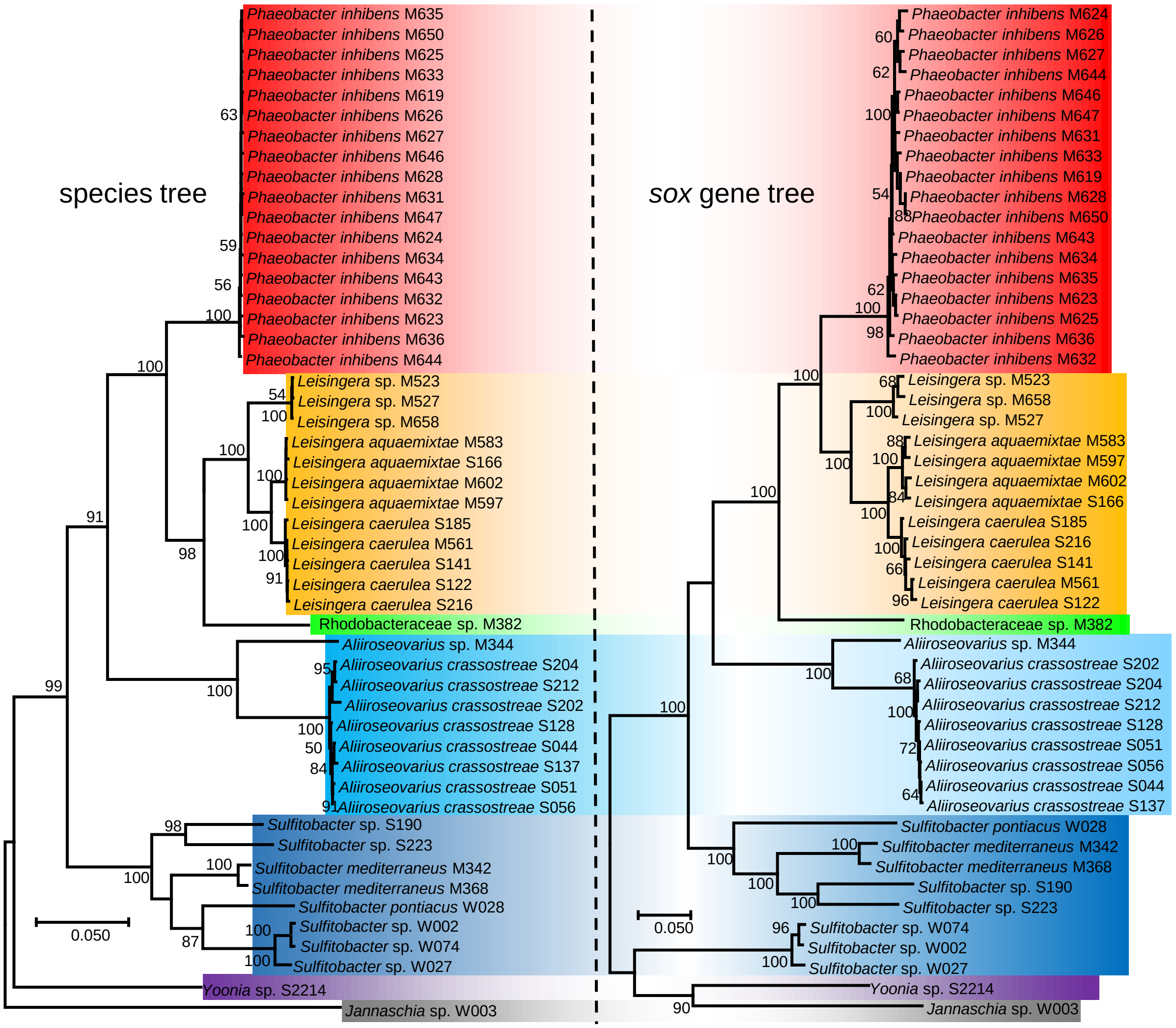

Supplementary Fig. 10 Comparison of the concatenated 31 single-copy genes tree and the concatenated *soxXYZABCD* genes tree for the biofilm *Roseobacter* strains. Trees were constructed using a maximum-like phylogency with 500 bootstrap samplings.

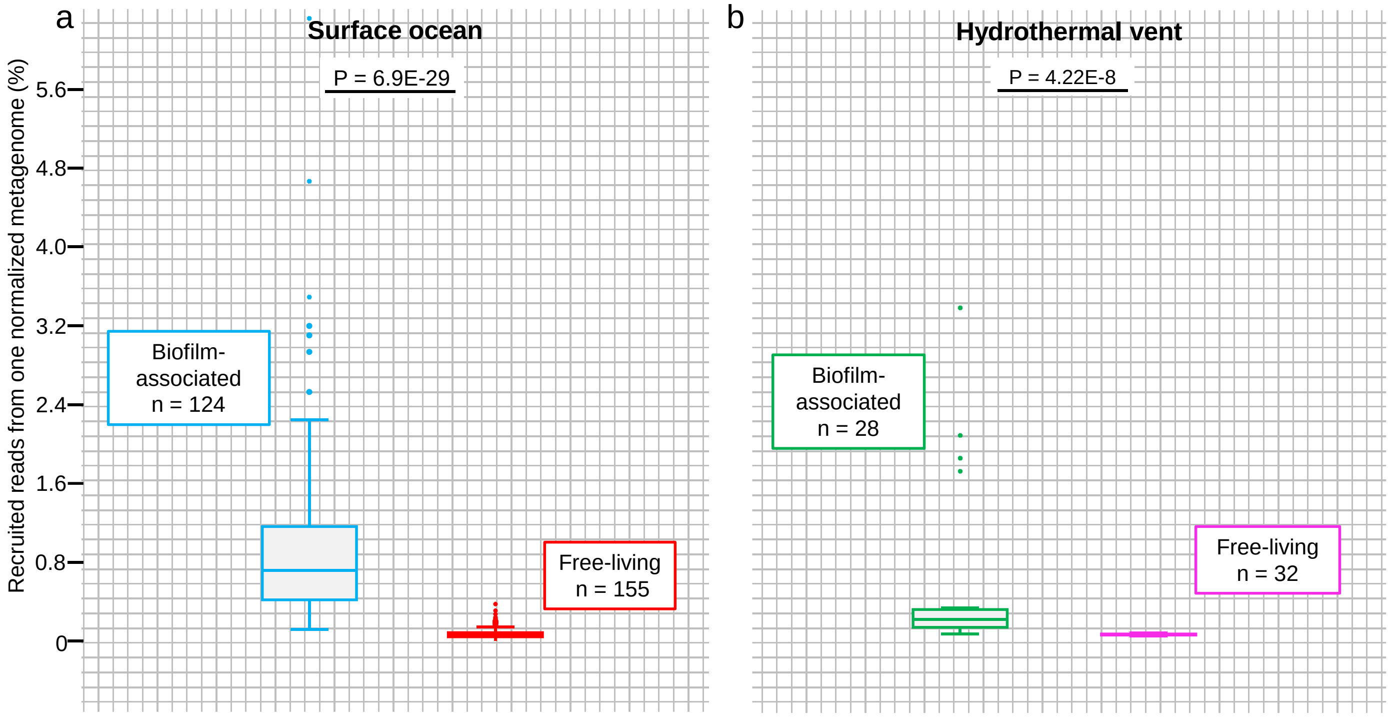

Supplementary Fig. 11 Summarized abundance of the biofilm *Roseobacter* strains in biofilm-associated and free-living microbiota collected across the global ocean. **a**, Comparision between surface ocean biofilms and seawater microbiota. **b**, Comparision between hydrothermcal vent biofilms and fluid microbiota. Total abundance of the 54 strains in a given metagenome is calculated by summarizing the recruited metagenome reads by the 54 respective genomes. All the metagenomes included for analyses were normalized to 1,000,000 reads with a read length of 101 bp. In all boxplots, central line represents the median, bounds represent upper and lower quartiles, whiskers represent maximum and minimum, and potential outliers are indicated by dots. P-values are derived from two-sided Students' t-test. Source data are provided as a Source Data file.

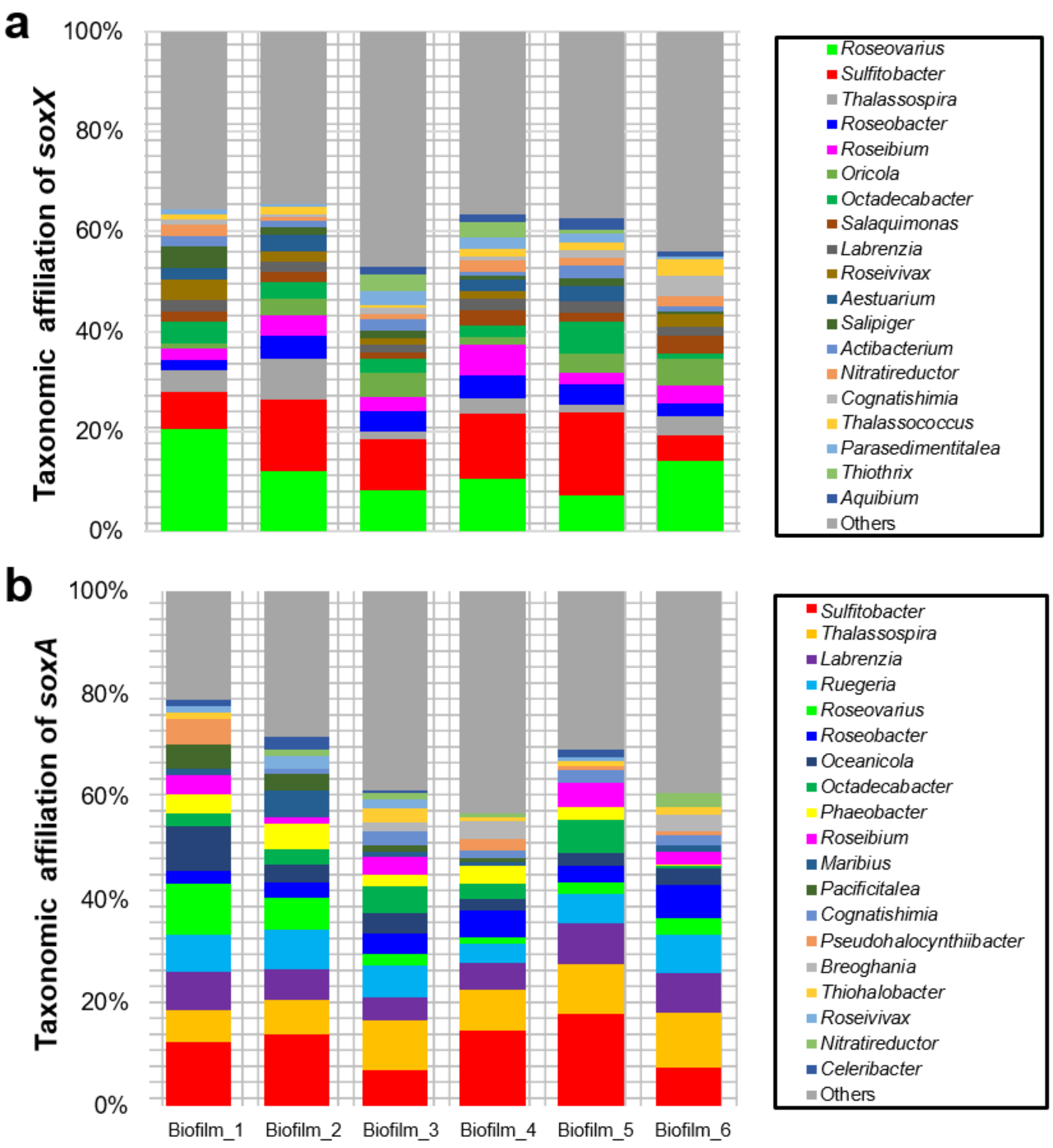

Supplementary Fig. 12 Taxonomic affiliation of the *soxX* and *soxA* genes in assembled biofilm metagenomes at genus level. The six biofilms collected in the present study were used for analyses. The affiliations were determined by searching against the NCBI-Nr database using Kaiju. **a**, *soxX*; **b**, *soxA*. Source data are provided as a Source Data file.

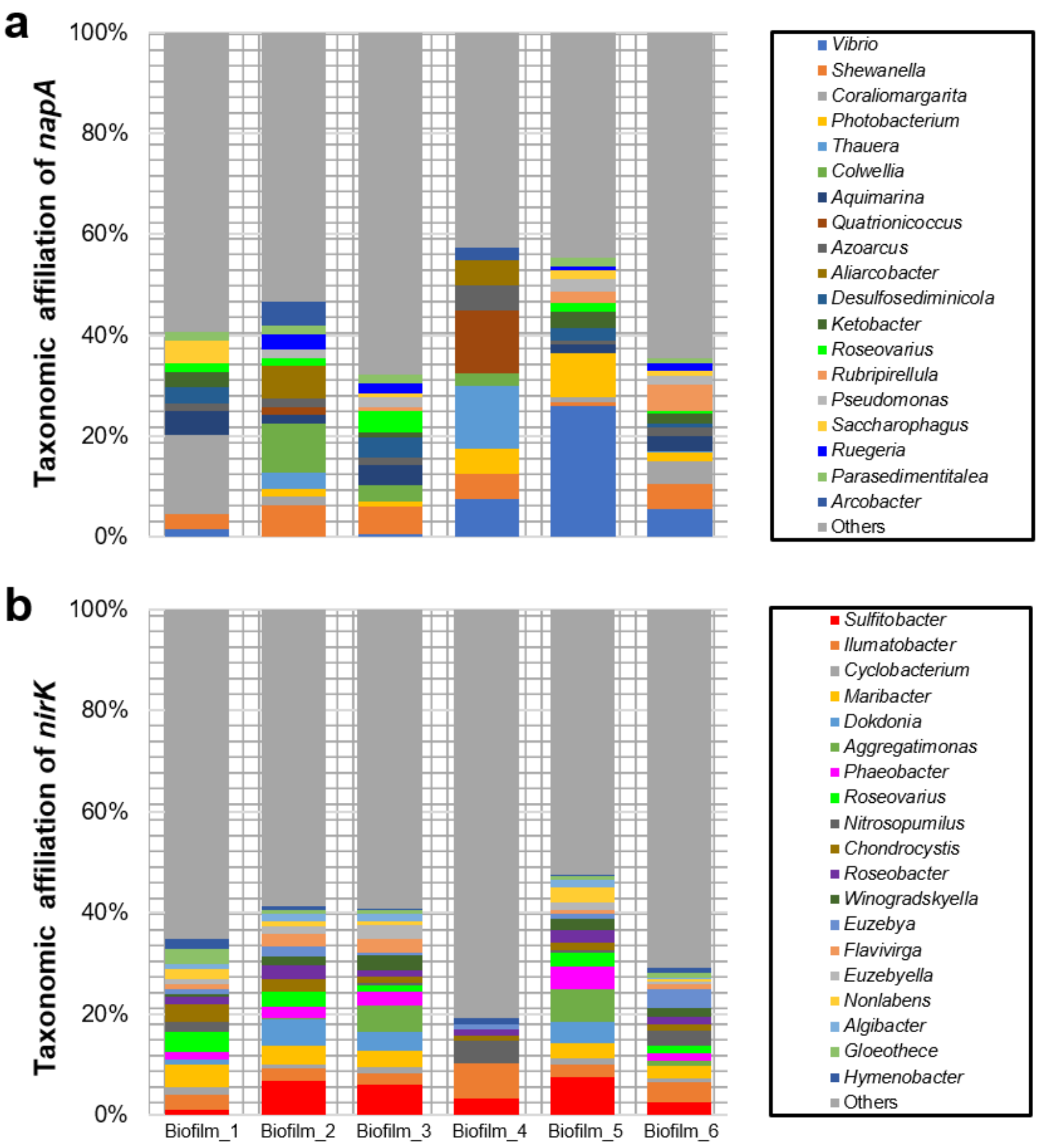

Supplementary Fig. 13 Taxonomic affiliation of the *napA* and *nirK* genes in assembled biofilm metagenomes at genus level. The six biofilms collected in the present study were used for analyses. The affiliations were determined by searching against the NCBI-Nr database using Kaiju. **a**, *napA*; **b**, *nirK*. Source data are provided as a Source Data file.

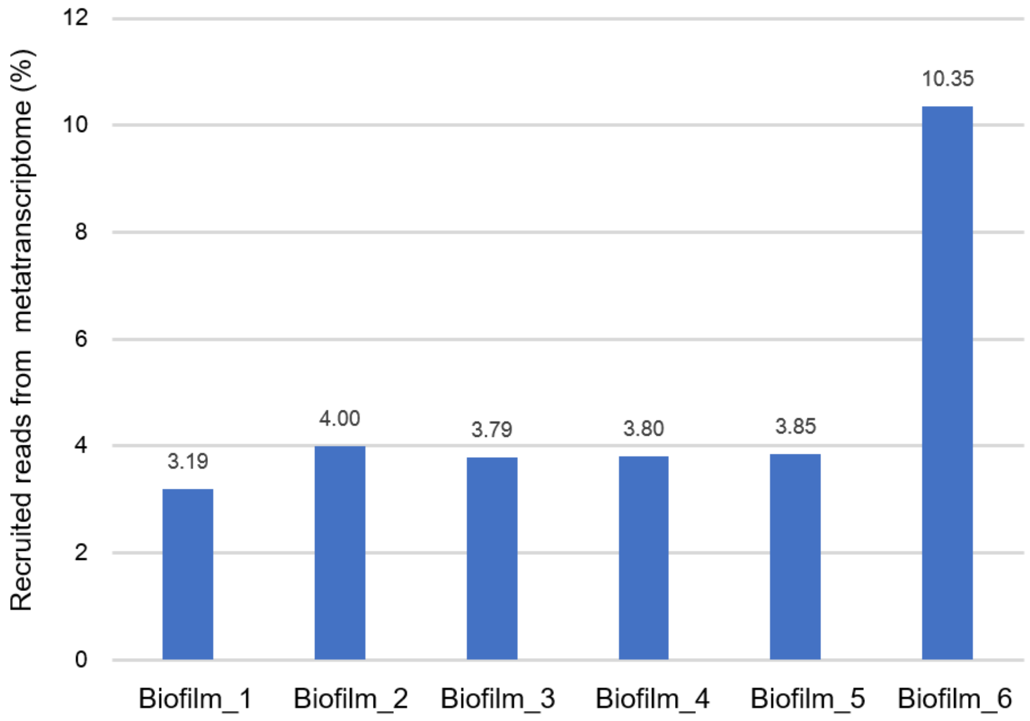

Supplementary Fig. 14 Summarized abundance of the biofilm *Roseobacter* strains in biofilm metatranscriptomes collected in different months. The accumulated percentage of the metatranscriptomic reads recruited by the chromosomoes of the 54 *Roseobacter* strains is shown.

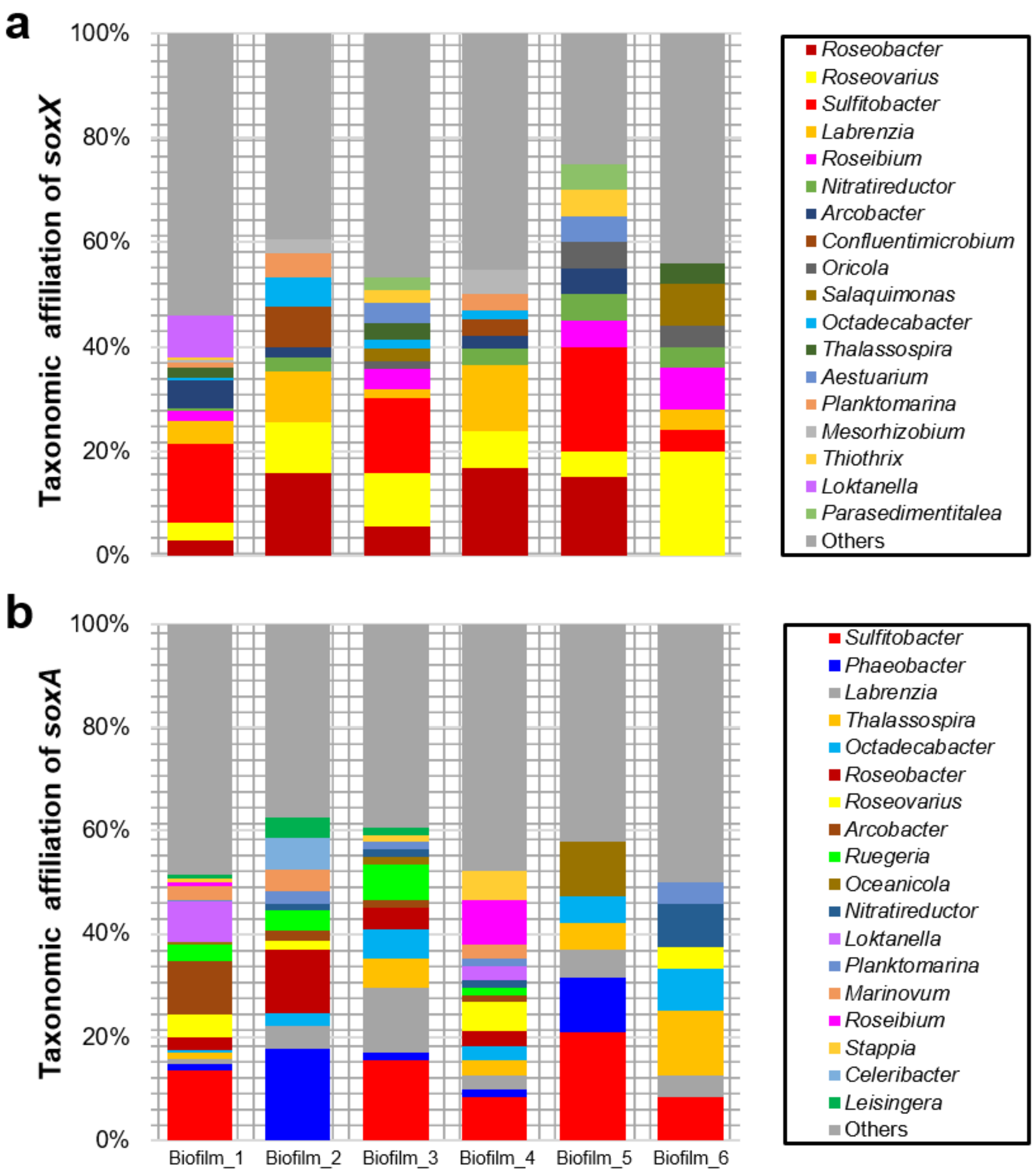

Supplementary Fig. 15 Taxonomic affiliation of the *soxX* and *soxA* genes in assembled biofilm metatranscriptomes at genus level. The six biofilms collected in the present study were used for analyses. The affiliations were determined by searching against the NCBI-Nr database using Kaiju. **a**, *soxX*; **b**, *soxA*. Source data are provided as a Source Data file.

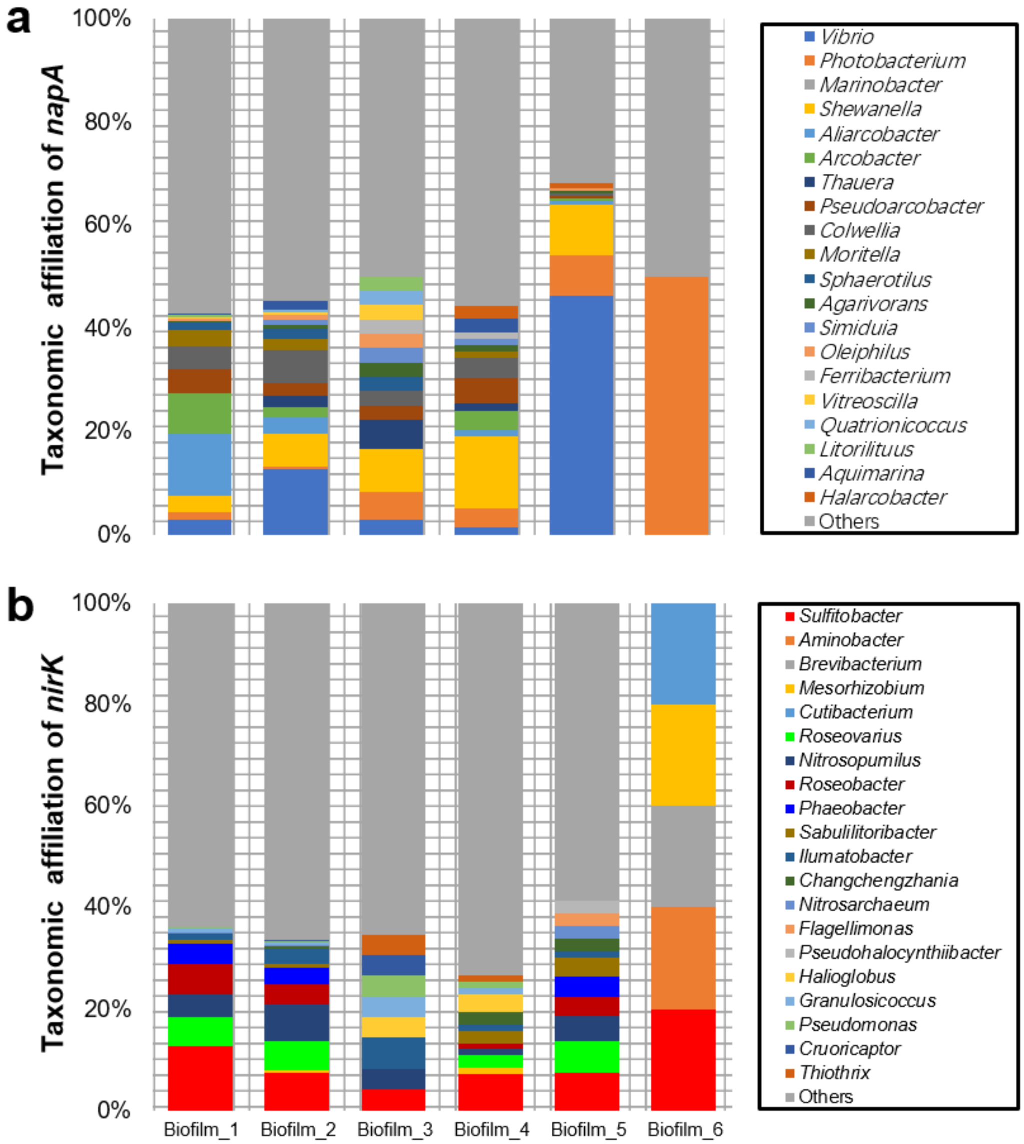

Supplementary Fig. 16 Taxonomic affiliation of the *napA* and *nirK* genes in assembled biofilm metatranscriptomes at genus level. The six biofilms collected in the present study were used for analyses. The affiliations were determined by searching against the NCBI-Nr database using Kaiju. **a**, *napA*; **b**, *nirK*. Source data are provided as a Source Data file.

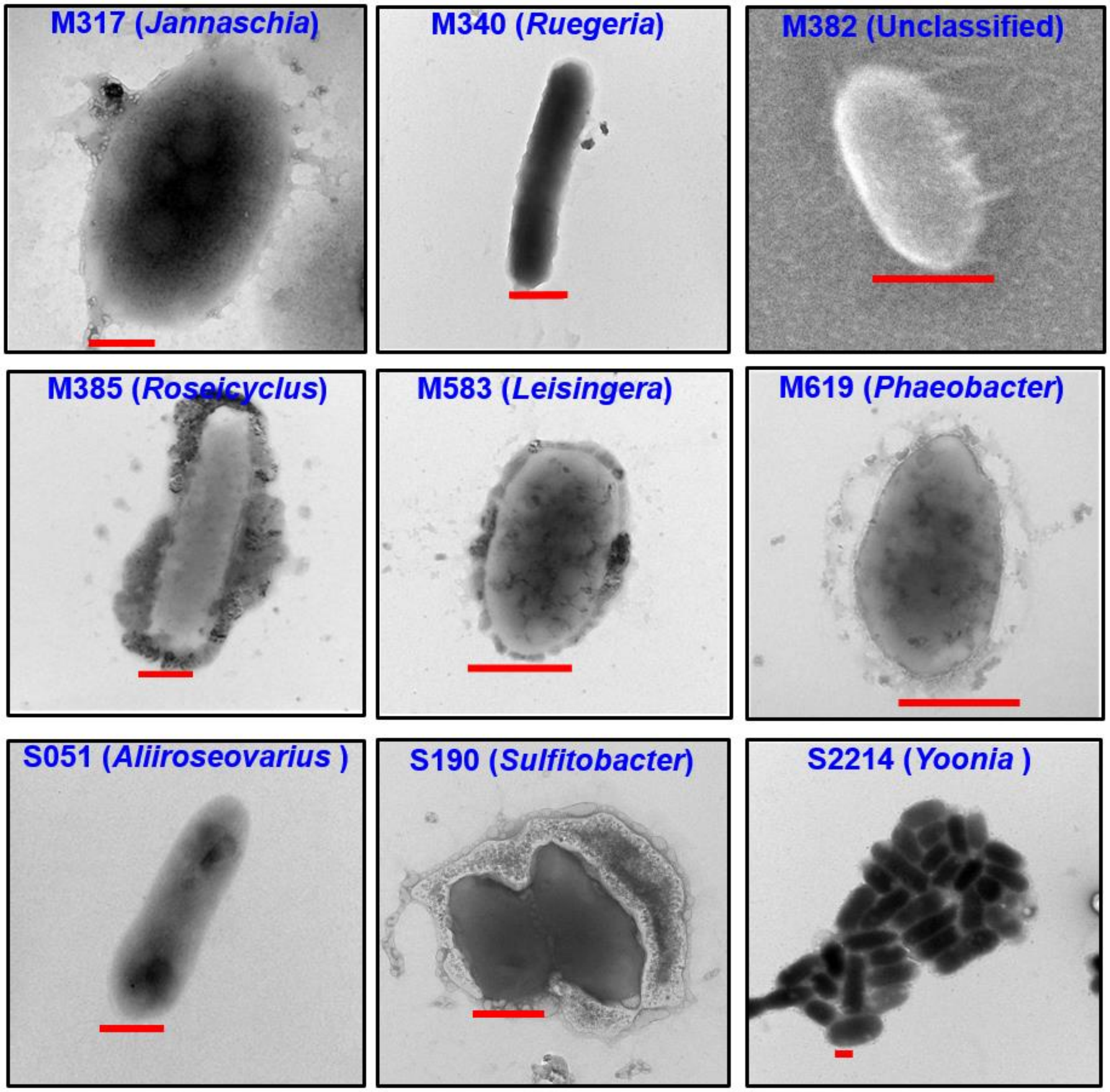

Supplementary Fig. 17 Transmission electron microscopy observation of selected biofilm *Roseobacter* strains. Nine strains from the nine distinct genera were observed at 10,000-50,000 times magnification. Scale bar = 500 nm.

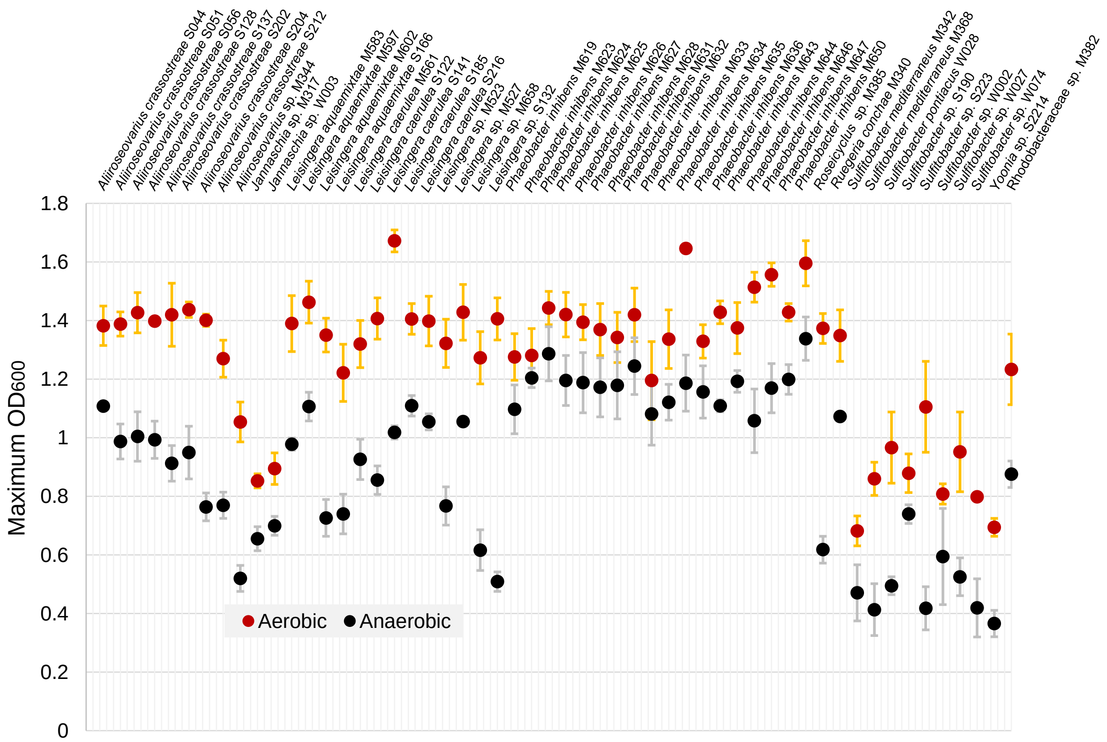

Supplementary Fig. 18 Maximum biomass of the 54 biofilm *Roseobacter* strains when grown in aerobic and anaerobic conditions. Strains were cultured in marine broth 2216 media at 25 ℃ and the biomasses were indicated by measuring optical densities at 600 nm wavelength (OD_{600}). Values are shown as mean ± s.d. (n = 3 biologically independent replicates). Source data are provided as a Source Data file.

.

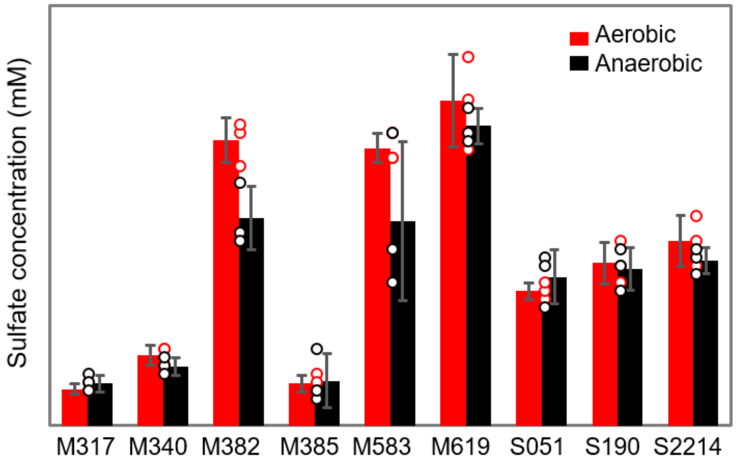

Supplementary Fig. 19 Thiosulfate oxidation by representative biofilm *Roseobacter* strains. Sulfate production in nine strains from the nine distinct genera were detected. Strains were grown in minimum media with 10 mM thiosulfate for 72 hours under aerobic and anaerobic conditions. In the bar charts, values are shown as mean ± s.d. (n = 3 biologically independent replicates). Source data are provided as a Source Data file.

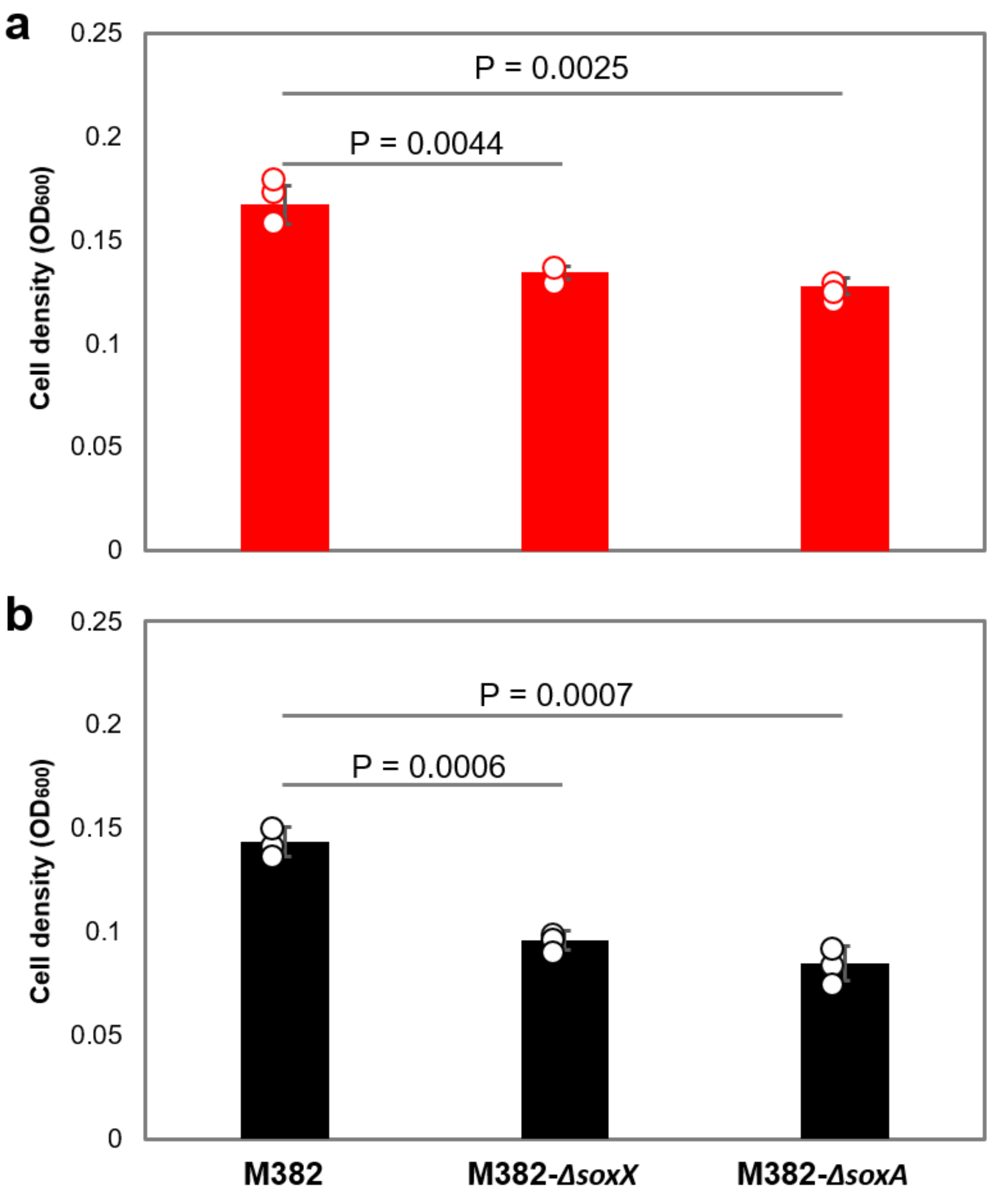

Supplementary Fig. 20 Growth of wild-type M382 and its two *sox* gene mutants in biofilms. The biofilms were grown in minimum media with 10 mM thiosulfate, followed by measurement of cell density at 600 nm after cultivation for 72 hours. Both aerobic (**a**) and anaerobic (**b**) conditions were studied. Two-sided Student's t-test was used to detect the significant difference. In the bar charts, values are shown as mean ± s.d. (n = 3 biologically independent replicates). Source data are provided as a Source Data file.

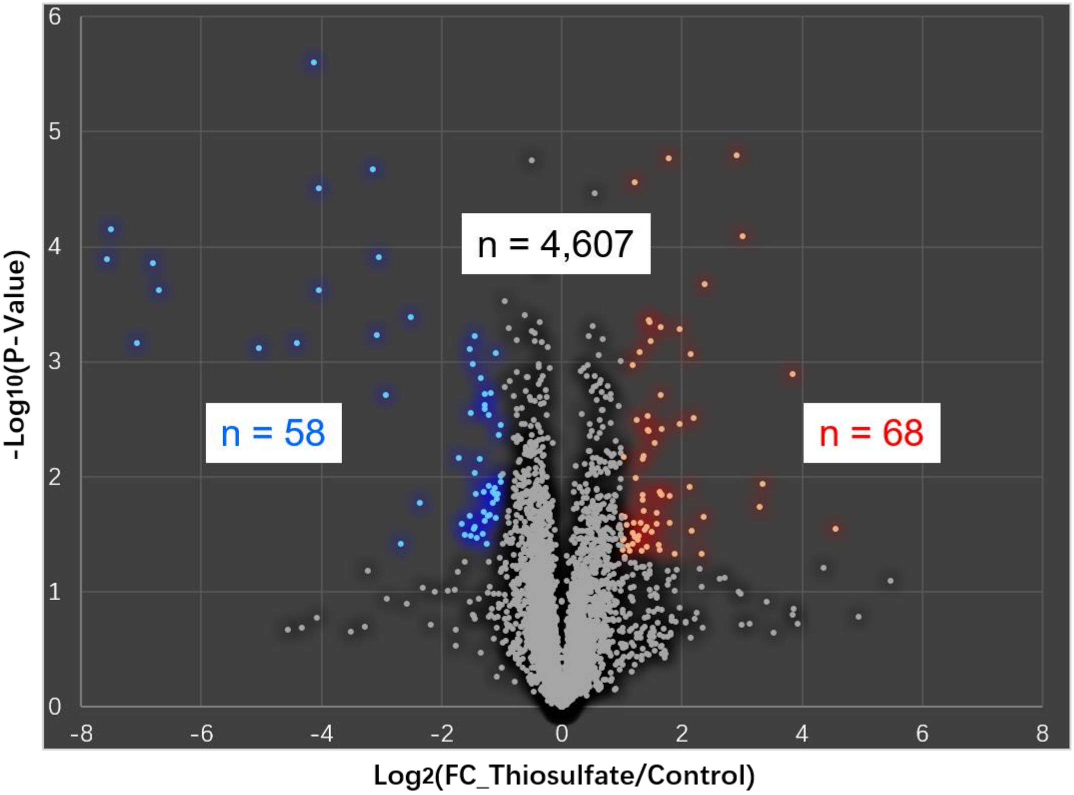

Supplementary Fig. 21 Overview of gene transcriptome profiles in M382 biofilms cultured with or without thiosulfate. Statistical analysis based on Reads Per Kilobase per Million mapped reads values was performed using two-sided Student's t-test with a threshold of fold change > 2 and P-value < 0.05. The up-regulated, down-regulated, and unchanged genes were colored in red, blue, and grey, respectively. Source data are provided as a Source Data file.

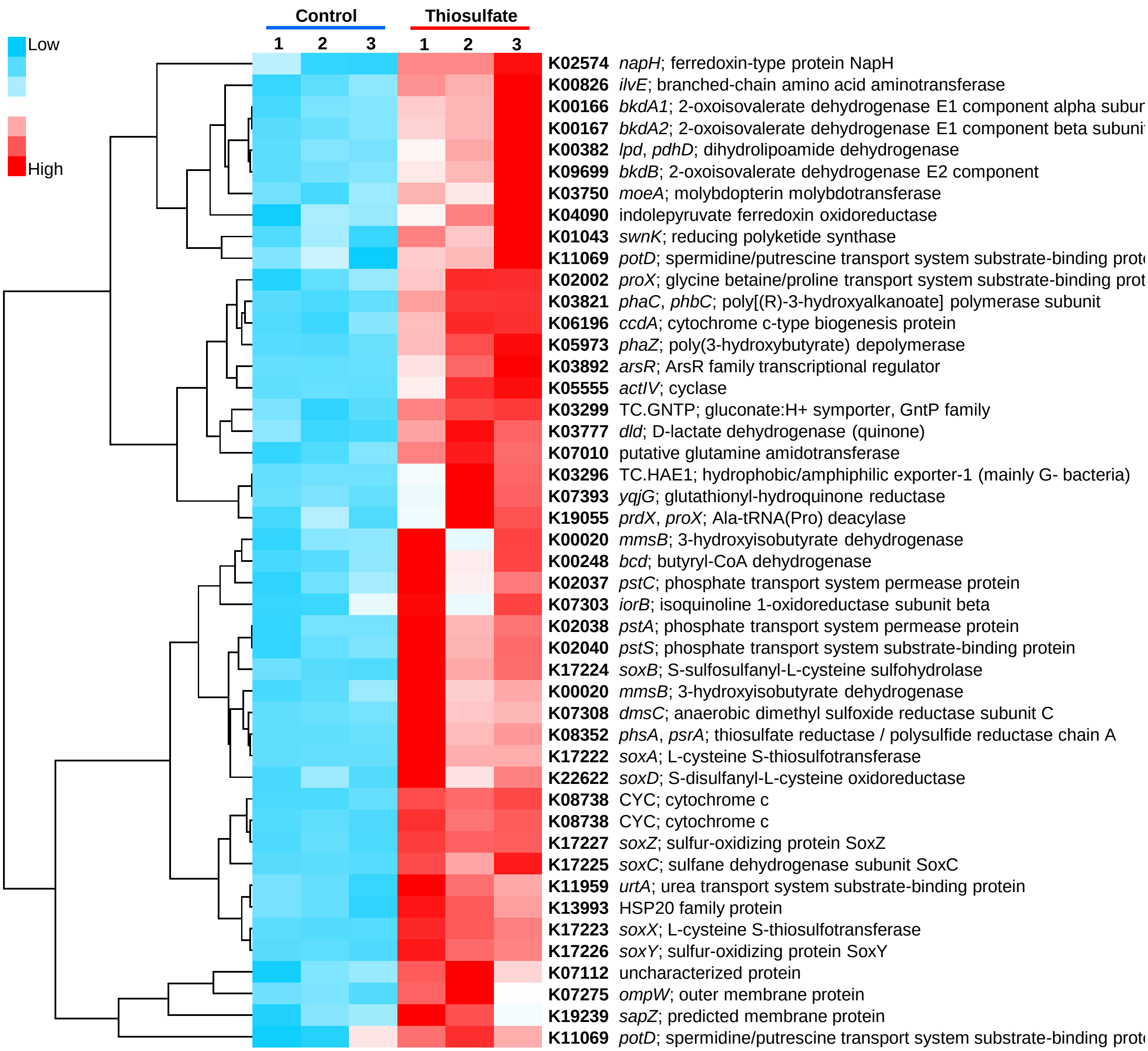

Supplementary Fig. 22 Functional annotation of the up-regulated genes in M382 biofilms by thiosulfate. Statistical analysis based on Reads Per Kilobase per Million mapped reads was performed using two-sided Student's t-test with a threshold of fold change > 2 and P-value < 0.05. Annotation was performed by searching against the KEGG database using E-value < $1e^{-5}$ and the annotated genes are shown. Source data are provided as a Source Data file.

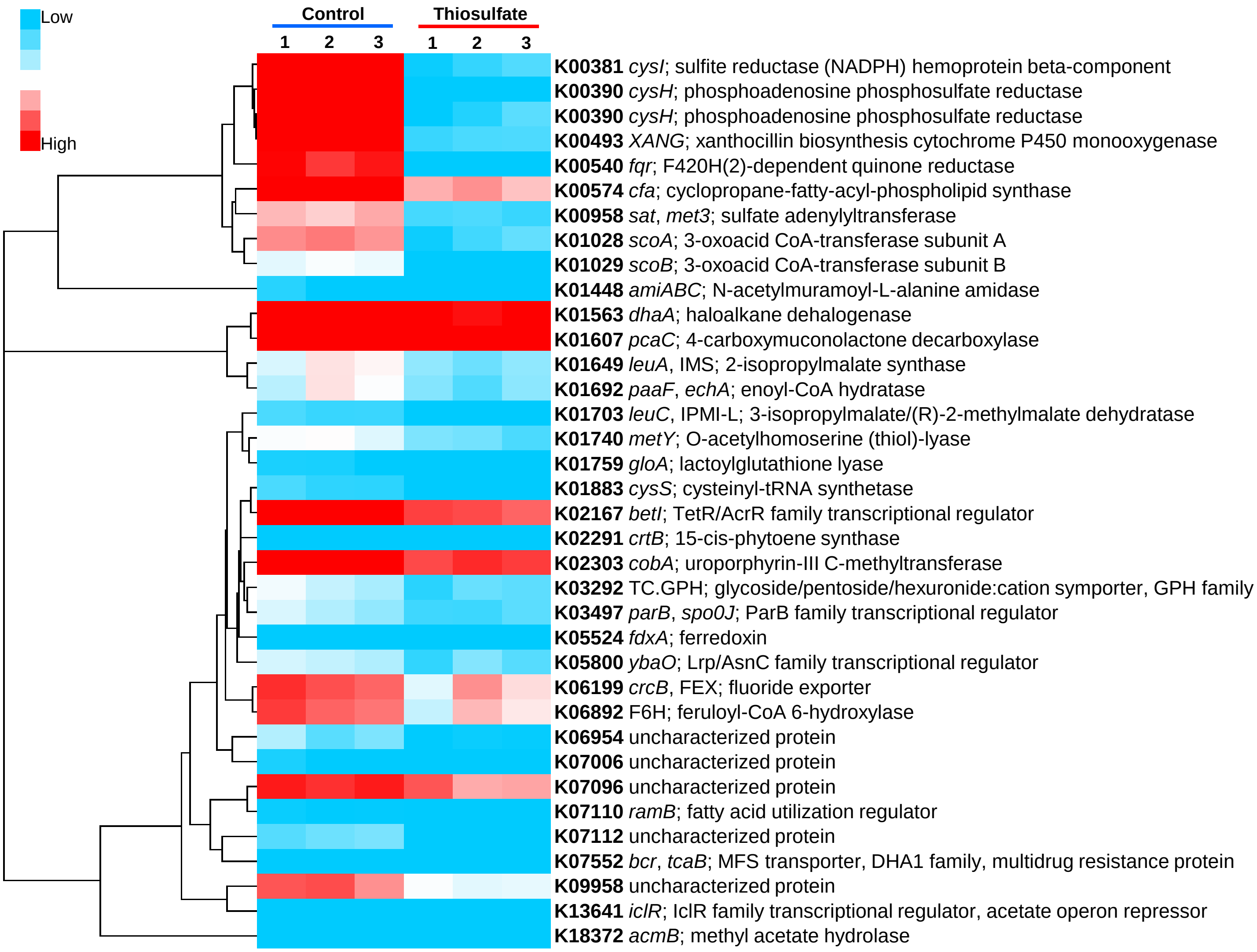

Supplementary Fig. 23 Functional annotation of the down-regulated genes in M382 biofilms by thiosulfate. Statistical analysis based on Reads Per Kilobase per Million mapped reads was performed using two-sided Student's t-test with a threshold of fold change > 2 and P-value < 0.05. Annotation was performed by searching against the KEGG database using E-value < 1e^{-5} and the annotated genes are displayed. Source data are provided as a Source Data file.

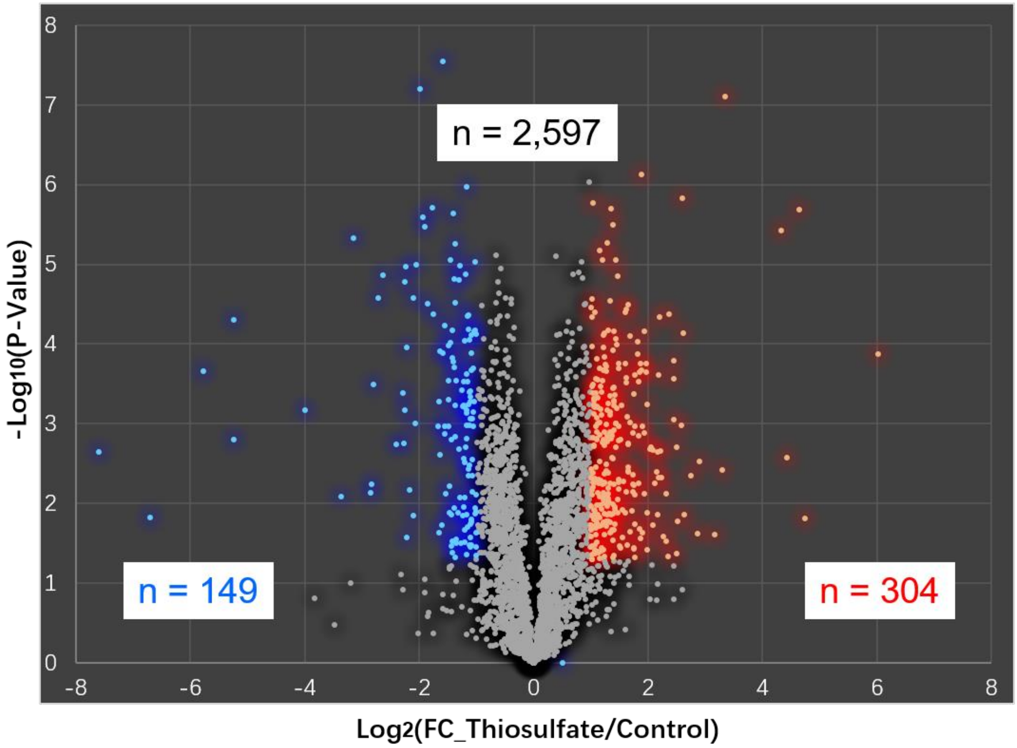

Supplementary Fig. 24 Overview of cell membrane proteome profiles in M382 biofilms cultured with or without thiosulfate. Statistical analysis based on label-free quantitation algorithm was performed using two-sided Student's t-test with a threshold of fold change > 2 and P-value < 0.05. The up-regulated, down-regulated, and unchanged proteins were colored in red, blue, and grey, respectively. Source data are provided as a Source Data file.

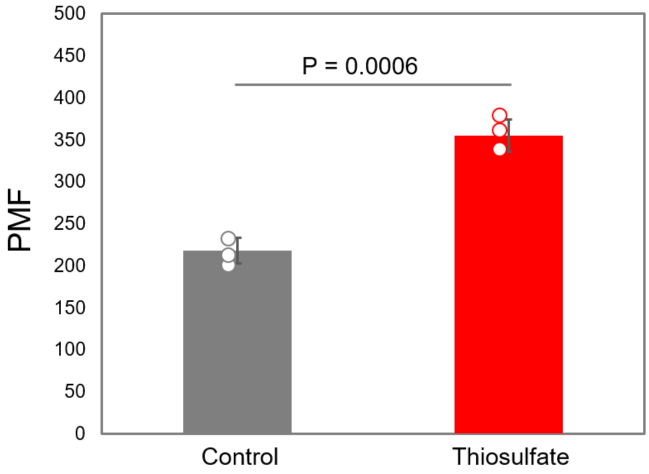

Supplementary Fig. 25 Proton motive forces (PMFs) in M382 biofilms grown with or without thiosulfate. The PMF was indicated by the fluorescence intensity of DiSC3(5). Two-sided Student's t-test was used to examine significant differences. In the bar charts, values are shown as mean ± s.d. (n = 3 biologically independent replicates). Source data are provided as a Source Data file.
