# Supplementary material for: Anaerobic thiosulfate oxidation by the Roseobacter group is prevalent in marine biofilms
Source: Nat Commun. 2023 Apr 11;14:2033. doi: 10.1038/s41467-023-37759-4 (PMC10090131; doi:10.1038/s41467-023-37759-4)
Supplement: Supplementary file 3 — Description of Additional Supplementary Files [file 41467_2023_37759_MOESM3_ESM.pdf]

Description of Additional Supplementary Files:

Supplementary Data 1: Complete genome features of the 54 strains isolated from coastal marine biofilms. The taxonomy is determined using GTDB-Tk classification.

Supplementary Data 2: Information of the metagenomes of biofilm-associated (n = 152) and free-living (n = 187) microbiota used for comparative analyses of the biofilm Roseobacter strains' distribution. In total six metagenomes were sequenced in the present study, 333 were downloaded from NCBI, including those reported in our previous studies and those from other research groups. The samples were collected from global surface ocean (biofilms on artificial surfaces, plastic surfaces, and plant root surfaces, and free-living microbes in seawater) and deep-sea hydrothermal vents (biofilms on artificial surfaces and chimney surfaces, natural microbial mats, and free-living microbes in vent fluids). # indicates unavailable.

Supplementary Data 3: Information of the assemblages of the six biofilm metagenomes sequenced in the present study.

Supplementary Data 4: Data information of the six biofilm metatranscriptomes sequenced in the present study.

Supplementary Data 5: Data information of the strain M382 transcriptomes sequenced in the present study.

Supplementary Data 6: Full list of the significantly changed membrane proteins in M382 biofilms treated versus untreated by thiosulfate.
